# Fingerprint Analysis of Buriti ( *Mauritia flexuosa* ) Using Paper Spray Mass Spectrometry

**DOI:** 10.1002/jms.5156

**Published:** 2025-07-05

**Authors:** Bruna V. Nunes, Ana Luiza C. C. Ramos, Talvane Coelho, Viviane D. M. Silva, Afonso Henrique de Oliveira Júnior, Ricardo Manuel de Seixas Boavida Ferreira, Rodinei Augusti, Raquel L. B. de Araújo, Julio Onesio‐Ferreira Melo

**Affiliations:** ^1^ Departamento de Alimentos, Faculdade de Farmácia Universidade Federal de Minas Gerais Belo Horizonte Minas Gerais Brazil; ^2^ Departamento de Ciências Exatas e Biológicas Universidade Federal de São João del‐Rei Sete Lagoas Minas Gerais Brazil; ^3^ LEAF‐Instituto Superior de Agronomia Universidade de Lisboa Lisbon Portugal; ^4^ Departamento de Química Universidade Federal de Minas Gerais Belo Horizonte Minas Gerais Brazil

**Keywords:** Brazilian savanna, buriti, chemical profile, PS‐MS, residues

## Abstract

A straightforward and efficient approach using paper spray ionization mass spectrometry (PS‐MS) was employed to detect fixed constituents in all anatomical parts of buriti, a native Brazilian fruit. This ambient mass spectrometry technique requires minimal sample preparation and was applied in both positive and negative ion modes. In total, 61 compounds were identified, predominantly in the negative mode, 26 of which had not been previously reported in the literature for this fruit. Although the pulp had been the sole focus of prior studies on fixed constituents, this work reveals novel findings for other fruit parts (peel, endocarp, and almond). Flavonoids emerged as the major phenolic compounds across all fractions, with the peel showing the highest compositional diversity. Given that existing literature focuses almost exclusively on the commercially exploited pulp—and considering the biological significance of the compounds identified here—this study demonstrates the potential for whole‐fruit utilization. Such an approach could not only add value to buriti‐derived products but also generate income for local producers and contribute to preserving the Cerrado biome.

## 1 Introduction

Among the biomes in Brazil, the Cerrado stands out for corresponding to 25% of the national territory [1, 2]. Fruits found in the Cerrado biome have peculiar characteristics, such as varied shapes, attractive colors, and unique flavors. In addition to the sensory characteristics that make their consumption attractive, these fruits stand out for being potential sources of vitamins and bioactive compounds, thus contributing to human health [3, 4, 5, 6, 7, 8]. The buriti fruit harvested from the buriti palm ( *Mauritia flexuosa* L.) is widely distributed in the Cerrado biome, particularly in flooded or humid areas of the Midwest, North, and Northeast regions [1, 2, 3].

**FIGURE 1:**
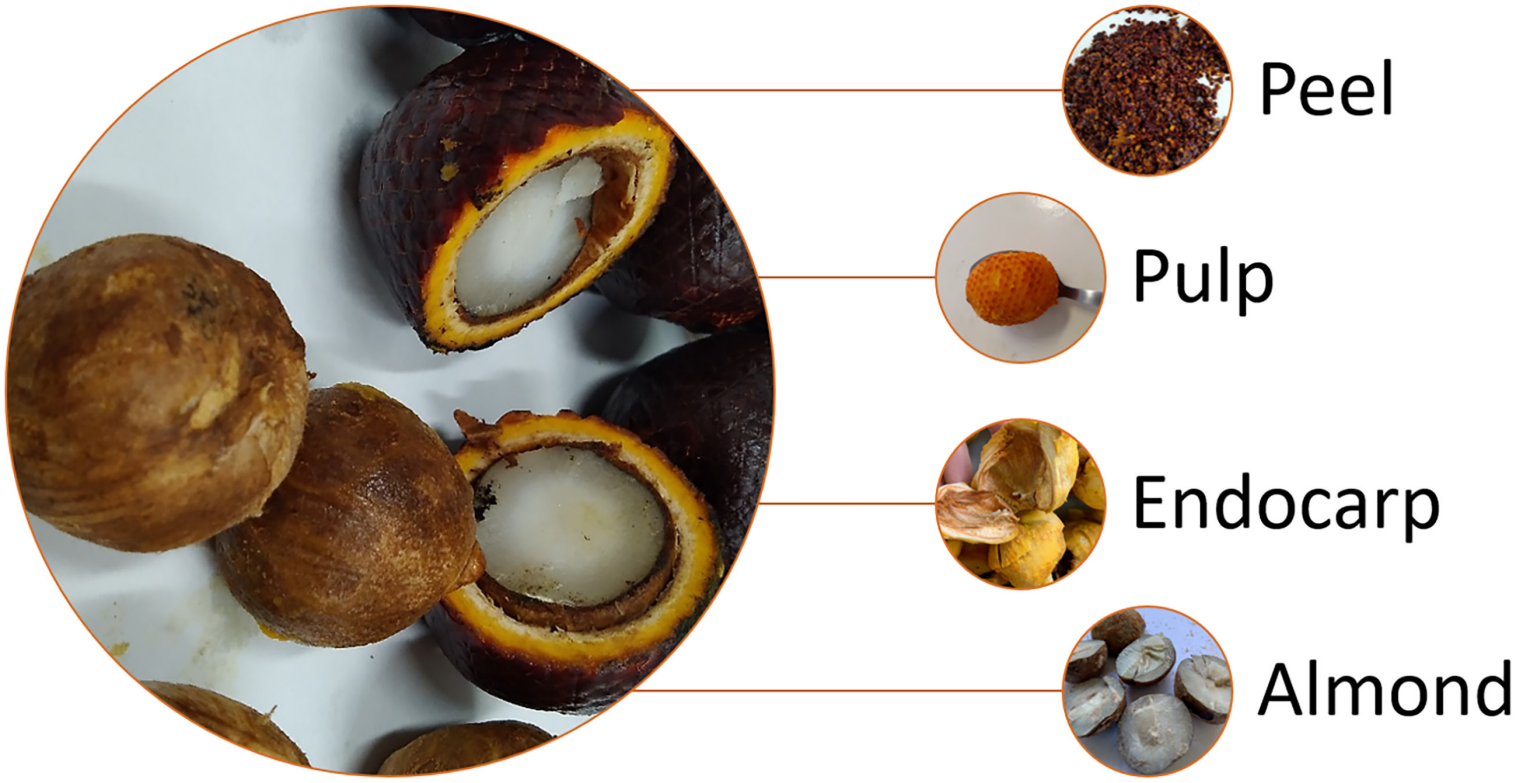
Parts of buriti **(** *M. flexuosa* **).**

The buriti palm ( *M. flexuosa* L.) features a straight, cylindrical trunk that can grow up to 35‐m tall. Its fruits are covered in reddish‐brown scales and contain a fresh, edible pulp that is orange, soft, and water soluble. Each fruit also houses numerous small, flat seeds (almonds), which are circular and range in color from red to brown [4, 5, 6]. The buriti fruit matures between March and August exclusively on female palms. The fruit exhibits an elliptical to oval shape and consists of four distinct anatomical layers: pericarp (peel), mesocarp (pulp), endocarp (lignocellulosic surrounding the almond), and endosperm (almond) (Figure 1).

Owing to its unique compositional diversity across different fruit structures, the buriti palm offers versatile applications across multiple industries. The plant serves as a valuable resource for food industry, cosmetics production, handicrafts, and biofuels [7, 8]. The buriti fruit's unique composition enables versatile uses: edible oils with vermifuge properties, cosmetics for burns and UV protection, and food products like juices, liquor, and ice cream [9, 10, 11, 12]. Additionally, it has been traditionally used to address respiratory ailments (pneumonia and flu), snake bites, and cardiovascular conditions [11, 13, 14, 15, 16].

Market demand for buriti continues to rise, with Brazil's North and Northeast regions harvesting an annual average of 470 metric tons (IBGE, 2021). However, current industrial processing focuses predominantly on the pulp, which constitutes just 18%–30% of the fruit's total mass, suggesting substantial untapped utilization potential [12, 17, 18, 19]. Other parts, such as the peel, endocarp, and almonds, are usually discarded or used for the production of handicrafts or animal feed [20, 21, 22]. It is estimated that buriti residues (peel, endocarp, and almond) reach about 18 tons per hectare cultivated each year [11].

Given buriti's potential as an alternative income source for local Cerrado producers and the significant waste generated during fruit processing, a deeper understanding of its chemical composition could expand marketable product ranges and add value to by‐products. UHPLC‐ESI/MS/MS, UHPLC‐MS/MS, and HPLC‐DAD‐ESI/MS techniques were employed to analyze buriti pulp [1, 23, 24]. Additionally, UPLC‐ESI‐MS/MS was used to evaluate extracts from the leaves, trunk, and whole fruit (including peel and pulp), whereas LC‐ESI‐MS/MS was applied to almond and peel extracts [25, 26]. Notably, all cited studies examined buriti fruits sourced from the Amazon regions of Ecuador, Peru, or Brazil.

Paper Spray Ionization Mass Spectrometry (PS‐MS) is a technique that has the advantage of fully direct, minimal sample preparation and solvent use, as well as high sensitivity and selectivity of the analytical signal for comprehensive natural product screening via PS‐MS [27, 28, 29, 30, 31]. PS‐MS (Paper Spray Mass Spectrometry) has been successfully employed for the rapid screening of various native Brazilian fruits, including baru [32], cagaita [33, 34, 35], araticum [36], grumixama [37], pêra do Cerrado [38], jabuticaba and jambolão [39], or geographical differentiation of varieties like coffee [29]. As such, considering that this technique has not yet been reported in the literature for the identification of phytochemicals in the buriti fruit, this study aims to investigate the fixed chemical constituents of the peel, pulp, endocarp, and almond of *M. flexuosa* from the Brazilian Cerrado biome, in the positive and negative ion modes.

## 2 Materials and Methods

### 2.1 Fruit Harvest and Selection

Fresh buriti fruits were harvested in November 2022 from the municipality of Três Marias, Minas Gerais, Brazil (17°49′16″ S 44°19′11″ W). Following standard practice, ripe fruits were collected from the ground after their natural fall peak.

At the Organic Chemistry and Phytochemistry Laboratory (Universidade Federal de São João del‐Rei, *campus* Sete Lagoas), fruits were selected based on optimal condition criteria: absence of dents, physical damage, and uniform appearance. Selected fruits underwent sanitization with sodium hypochlorite (0.1% *w*/*v*) followed by manual fractionation into four anatomical parts: peel, pulp, endocarp, and almond. Pulp and endocarp were homogenized using an analytical mill (IKA A11 Basic, United States). Peel was mechanically crushed, and almond was flaked with a surgical‐grade steel knife due to its hardened structure. All fractions were immediately packaged in airtight plastic containers, frozen at −18°C, and protected from light exposure during storage and transport in thermal boxes to the Mass Spectrometry Laboratory (Department of Chemistry, Federal University of Minas Gerais, campus Pampulha).

### 2.2 Obtaining the Extracts

On an analytical scale, 1.0 g of each part of the buriti fruit was weighed in 15‐mL Falcon tubes and processed, and 8 mL of HPLC‐grade methanol (Sigma, Supelco) was added. Samples were then vortexed for 30 s and kept at rest at room temperature (25°C) until the chemical profile analysis by PS‐MS was carried out Ramos et al. [40].

### 2.3 Chemical Profile Analysis

Extracts of the buriti parts were analyzed by PS‐MS in a Thermo LCQ‐Fleet mass spectrometer (ThermoScientific, San Jose, United States) in the positive and negative ion modes. The PS source was assembled according to the methodology described by Ramos et al. [40]. From the extracts of the previously prepared samples, 2 μL was collected and placed on the base of a triangular‐shaped chromatography paper (1.0 × 1.5 × 1.5 cm), followed by the addition of methanol (40 μL). Instrumental conditions used were 4.5‐kV paper voltage; capillary temperature of 275°C; 40‐V capillary voltage; 120‐V tube lens voltage; 100–1000 *m/z* range for full scanning; and 15‐to‐45‐unit collision energy for ion fragmentation. Processing of the mass spectra data was performed using Xcalibur Version 2.1 (Thermo Scientific, San Jose, CA, United States). Excel 2020 (Microsoft, Redmond, WA, United States) was used to list and organize the average mass spectra for further analysis. Metabolites were tentatively identified by comparing their masses and fragmentation patterns with those described in the literature.

## 3 Results and Discussion

**FIGURE 2:**
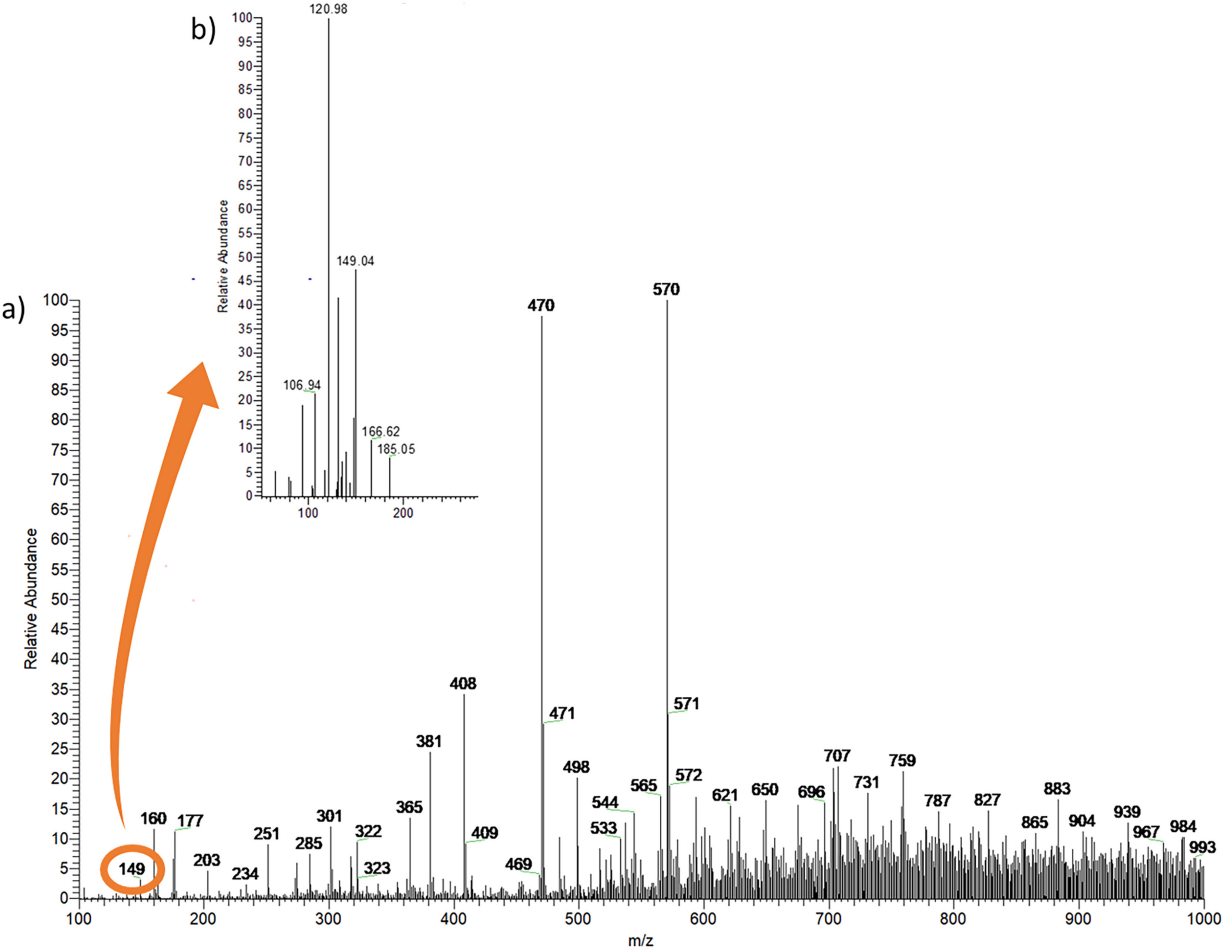
(a) Positive‐ion mode paper spray mass spectrum (PS (+) MS) of the buriti almond methanolic extract and (b) tandem mass spectrum (MS/MS) of the precursor ion at *m*/*z* 149 ([M + H] ^+^), putative cinnamic acid.

Figure 2 illustrates the workflow for ion identification. Figure 2a shows the full‐scan mass spectrum of the buriti almond methanolic extract, from which the 200 most intense peaks were selected. These ions were cross‐referenced with literature reports for the same botanical family (Arecaceae). When tentative identifications were made based on fragmentation patterns, targeted MS/MS analysis was performed. The experimental MS/MS spectra were then compared with published fragmentation profiles for confirmation.

As shown in Table 1, 34 different chemical compounds were identified in the buriti fruit in the negative ion mode, with 28 phytochemicals found in the peel, 24 in the endocarp, 17 in the pulp, and only 3 in the almond. As for the positive mode, as shown in Table 2, 15 compounds were found, most of which were present in the peel of the fruit (9), followed by the endocarp (8), pulp (2), and almond (1). Regarding the chemical class of the compounds, there is predominantly the presence of flavonoids and phenylpropanoids, both in the positive and negative ion modes. In the positive ionization mode, fatty acids such as palmitic, oleic, and stearic acids were also detected. In the negative ion mode, the presence of other classes was detected, such as organic acids, coumarins, stilbenes, terpenoids, sugars, and benzoic acid derivatives.

**TABLE 1:** Chemical profile of the buriti fruit in the negative ion mode PS (−) MS.

Compound	Precursor ion (*m/z*)	Fragments (MS/MS)	Buriti fruit part				
			Peel	Endocarp	Pulp	Almond	Reference
Organic acids							
Furoic acid	111	67	X	X	X		[41]
Malic acid	133	71, 89, 115		X	X		[41]
Isopropylmalic acid	175	85, 115			X		[41]
Citric acid	191	87, 111	X	X	X		[41]
Quinic acid	191	85, 93	X	X	X		[42]
Cumarina (umbelliferone)	161	133	X				[26]
Phenylpropanoids							
Caffeic acid	179	89, 107, 135	X	X			[1, 24, 26, 42]
Ferulic acid	193	134	X	X	X		[26, 42]
5‐O‐Caffeoyl shikimic acid (neodactylifric acid)	335	179	X				[43]
Caffeic acid hexoside	341	161, 179	X	X	X		[25]
Chlorogenic acid	353	173, 179, 191	X	X	X		[1, 24, 25, 26, 42]
Schaftoside	563	353 383 443 473 503	X	X			[44]
Stilbenes							
Resveratrol	227	185				X	[24]
Pterostilbene	255	240	X	X			[24]
Flavonoids							
Pinocembrin	255	151	X	X			[26]
Naringenin	271	119, 151, 227	X				[1, 25, 26]
Kaempferol	285	257	X				[25, 26, 42]
Hispidulin	299	284	X	X	X		[26]
Hesperetin	301	286	X				[24]
Quercetin	301	151, 179, 272	X				[1, 24, 26, 42]
Isoquercetrin acetate	505	463	X	X	X		[43]
Kaempferol‐3‐O‐sulfate‐4′‐O‐*α*‐rhamnosyl (1 → 6)‐*β*‐d‐glucoside	673	593	X	X	X	X	[44, 45]
Monohexosides isomers of chrysoeriol	461	299	X				[46]
Kaempferol‐3‐*O*‐glucuronide	461	315	X				[1]
Catechin/epicatechin dimer	577	289 407	X	X	X		[41]
Luteolin‐7‐*O*‐derivatives	593	285, 447	X				[45]
Kaempferol‐3‐*O*‐derivative	593	284, 447	X				[45]
Cyanidin‐3‐rutinoside	595	449	X		X		[1, 25]
Rutin	609	271, 300		X			[24, 26]
Quercetin‐3‐O‐derivatives		300, 301					[45]
Isorhamnetin derivative	623	315, 459		X			[41, 45]
Quercetin‐dihexoside	625	301	X	X			[1, 45]
Isorhamnetin‐3,4′‐di‐O‐hexoside	639	315, 477		X			[45]
Quercetin‐3‐O‐(2‐sulfate, 6‐rhamnosyl) hexoside	689	609	X	X			[45]
Isorhamnetin‐3‐(2R, 6‐malonyl) hexose	709	665	X	X	X		[45]
Quercetin‐3 (2‐malonyl, 3/4 hexose) hexose	711	667	X	X	X		[45]
Quercetin‐3‐*O*‐(6‐rhamnosyl)	771	609	X	X	X		[45]
Isorhamnetin‐3‐*O*‐(6‐hexosyl) hexoside‐7‐O‐hexoside	801	639	X	X	X		[45]
Benzoic acid derivatives							
Ellagic acid	301	145, 151, 179, 229, 257, 273	X				[25, 26]
Terpenoids							
Bilobalide	325	163	X	X		X	[47]
Carnosol	329	285	X	X	X		[26, 47]
Sugars							
Sucrose	341	119, 179	X	X	X		[41]

Abbreviation: X, identified.

**TABLE 2:** Chemical profile of the buriti fruit in the positive ionization mode PS (+) MS.

Compound	Precursor ion (*m*/*z*)	Fragments (MS/MS)		Buriti fruit part			Reference
			Peel	Endocarp	Pulp	Almond	
Phenylpropanoids							
Cinnamic acid	149	107, 117, 121, 131	X	X	X		[48]
3‐O‐Caffeoylshikimic acid (dactylifric acid)	337	271		X			[47]
Fatty acids							
Oleic acid	282	97, 111, 264	X	X			[23]
Stearic acid	284	83	X	X	X		[23]
Flavonoids							
Catechin	291	139		X			[47]
Gallocatechin (−)	307	139		X			[47]
5Deoxyleuco pelargonidin	313	95, 109, 123, 137, 239, 257	X	X			[48]
Malvidine	331	315	X	X			[47]
Rhamnosyl hexosyl luteolin	595	449, 287	X			X	[49]
Apigenin di‐C‐hexoside	595	25	X			X	[49]
Pelargonidine	595	271	X			X	[47]
Apigenin caffeoyl hexoside	595	577, 339, 313, 539, 535, 357	X			X	[50]
Cyanidin‐3‐ rutinoside	595	449, 287	X			X	[1, 25]
Rhamnosyl hexosyl methyl luteolin	609	301, 463		X	X		[49]
Rhamnosyl exosyl quercetin	611	303, 465	X	X	X		[49]
Dihexosyl luteolin sulfate	691	287, 529	X		X		[49]
Rhamnosyl dihexosyl luteolin	757	611		X	X	X	[49]
Rhamnosyl dihexosyl methyl luteolin	771	608	X	X	X		[49]

Abbreviation: X, identified.

Flavonoids protect plants from ultraviolet (UV‐B) radiation and are relevant to human health, acting as anti‐inflammatory, antimicrobial, antitumor, and antiasthmatic agents [51]. These metabolites are associated with the fitness of plants and their ability to cope with environmental conditions and provide defense against herbivores, microorganisms, and other pathogens. In fruits and vegetables, these metabolites play relevant roles both in human health, due to their biological activities, and in influencing the color, flavor, and nutritional characteristics of foods [52].

Out of the 58 compounds identified in the buriti fruit by PS‐MS, 65.5% were flavonoids, such as quercetin and its derivatives. Quercetin is one of the most explored flavonoids due to its abundant presence in nature and in the human diet, as well as its marked biological activity, among which its antioxidant capacity stands out [53]. In addition to other biological effects that have also been reported, such as anti‐inflammatory, antiulcer, antiallergic, antiviral, gastroprotective, antihypertensive, immunomodulatory, and anti‐infective effects [54].

Quercetin (*m/z* 301) and its derivatives identified tentatively in this study were detected in the peel, endocarp, and pulp. Tauchen et al. [24], when studying the phenolic content of the plant extracts of the peel of *M. flexuosa* by UHPLC‐MS/MS, observed that quercetin and its derivatives (isoquercitrin, quercetin‐3‐arabinoside, and rutin) were among the most predominant constituents in the analyzed extracts. The authors positively correlated phenolic content with antioxidant potential, suggesting that phenolics were the main constituents responsible for the antioxidant effect observed in this species of the Arecaceae family. Among the 14 phenolic compounds identified by Abreu‐Naranjo et al. [1] in the buriti pulp, quercetin glycosides were also the most predominant.

In addition to the glycosylated forms of quercetin, important flavonoids have been identified, mostly in the buriti peel, such as kaempferol and its glycosides. Kaempferol has been linked to increased antioxidant defense against free radicals and its ability to modulate different intracellular signal transduction pathways linked to apoptosis, angiogenesis, inflammation, and metastasis [55]. Similarly to quercetin, the glycoside conjugates in kaempferol appear to increase its bioavailability in humans. Once absorbed, kaempferol is transformed by hepatic metabolism and released into the bloodstream as methyl metabolites, glucuronide, and sulfate, which have a direct influence on the biological effects attributed to it.

These findings are in line with [26], which observed the presence of flavonoids in buriti peel and almond extracts, such as hispidulin (*m/z* 299), kaempferol (*m/z* 285), naringenin (*m/z* 271), quercetin (*m/z* 301), rutin (*m/z* 609), and pinocembrin (*m/z* 255). The authors concluded that the peel extract presented higher total phenolic content and antioxidant performance in relation to the almond extract, showing that it is possible to add value to this buriti fruit residue. In this study, narigenin was identified only in the peel, hispidulin in all parts of the whole fruit except almond, and pinocembrin was detected in the skin and, for the first time, in the endocarp. According to Koolen et al. [25], the presence of polyphenols in the trunk, leaf, and fruit extracts without almonds of *M. flexuosa* contributes to a potential commercial application of the buriti fruit as an economic natural antioxidant, in addition to the use of other parts of the palm tree.

In a study by Hoffmann et al. [41], phenolic compounds identified in the butia (*Butia* spp.) fruit, a fruit of the buriti family, such as rutin and isorhamnetin, were described as significant markers for discrimination of geographic origin and species. These flavonoids identified in this work in the negative ion mode are predominantly in the endocarp of the buriti fruit. Furthermore, in the study of the authors, there was the presence of luteolin, pinocembrin, and hesperetin in the butia fruit. Luteolin is reported to have high antioxidant, anti‐inflammatory, and antimicrobial activity [56]. de Oliveira et al. [57] identified the presence of luteolin in leaves of *M. flexuosa* , whereas Bataglion et al. [42] and Nonato et al. [58] found this compound in the buriti fruit pulp. In this study, in addition to the buriti fruit pulp, luteolin derivatives were also detected for the first time in buriti almonds and peel.

The *m/z* 335 ion, identified in this study only in the buriti fruit peel, was proposed by Farag et al. [43] as being the flavonoid acid 5‐O‐caffeoyl shikimic acid (neodactylic acid), considered one of the main phenolic acids in fruits of immature date palms ( *Phoenix dactylifera* ). According to the authors, these derivatives formed by the condensation of hydroxycinnamic acids with quinic or shikimic acid are phenolic compounds common in many plant families, contributing to their flavor. This compound had not yet been reported for the buriti fruit peel until this study.

In the leaf, trunk and fruit extracts of *M. flexuosa* , caffeic acid (*m/z* 179), and chlorogenic acid (*m/z* 353) were reported by Koolen et al. [25]. Considering the fruit without almonds, this result confirms what was found for chlorogenic acid, which, in the present study, was detected in the peel, pulp, and endocarp of the buriti fruit. Rudke et al. [26] found chlorogenic and ferulic acids (*m/z* 193) in the peel and almond extracts of buriti. Bataglion et al. [42] identified chlorogenic, ferulic, and caffeic acids in the buriti fruit pulp. Therefore, this is the first study to report the presence of ferulic acid in the endocarp of the buriti fruit.

Among the phenylpropanoids, schaftoside fragment ions (*m/z* 563) were identified by El‐Akad et al. [44] in the extract of the leaves of *Caryota mitis* , whereas in the present study, they were found in the peel and endocarp of the buriti fruit, both species belonging to the Arecaceae family. This type of C‐glycosyl flavone has been reported to have antimelanogenic activities [59], antineuroinflammatory drugs [60], and hepatoprotective effects, also being traditionally used to treat hepatitis, liver cirrhosis, and gallstones [61].

Fingerprinting of date palm fruit ( *P. dactylifera* ) indicated flavonols (rutin and isoquercetrin) and sugars (glucose, fructose, and sucrose) as biomarkers contributing to date palm fruit classification [43]. Sucrose (*m/z* 341), a compound revealed in this study for the buriti fruit peel, pulp, and endocarp, is an energy source and a signaling molecule in plants [62].

Hoffmann et al. [41] used LC‐MS to differentiate *Butia spp* (Arecaceae) and provide biomarkers for discrimination of diverse species: *B. catarinensis*, *B. odorata* , *B. paraguayensis*, and *B. yatay*. In addition to sugars, organic acids were associated with the degree of ripeness of the fruit and identified as markers of *Butia* spp. Organic acids have a strong sensory influence on fruits and vegetables, normally responsible for their sour taste, and are important at the cellular level with roles in energy production and the formation of precursors for amino acid biosynthesis and at the whole plant level in modulating adaptation to the environment [63].

de Souza [64] associated the highest concentrations of quinic acid with the higher antioxidant activity of the buritirana fruit (*Mauritiella armata*) without almonds. Among the organic acids identified in this study, furoic, malic, isopropimaalic, and citric acids had not yet been reported for the buriti fruit. Only quinic acid had already been described by Bataglion [42] and Lahlou [47] in the buriti fruit pulp, being presented for the first time in the peel and endocarp of the fruit.

Malic acid, already detected in fruits, can stimulate metabolism and increase energy production [65]. Participation of malic acid as an intermediate in CO_2_ fixation has been extensively studied in relation to the efficiency of water use because it allows plants to survive in arid regions. In addition to malic acid, citric acid production can play a role as a CO_2_ reservoir in certain plants [63].

Presence of citric acid is associated with a factor in the quality of the fruit's flavor [40]. This organic acid also participates in several physiological and biochemical processes, such as respiration, synthesis of primary and secondary metabolites, and postharvest shelf life [66]. In addition, it is widely used in the food, pharmaceutical, and beverage industries as an acidifying agent and flavor enhancer [67].

Rudke et al. [26] identified umbeliferone (*m/z* 161), a coumarin, in the buriti fruit peel extract using LC‐ESI‐MS/MS in negative ion mode. These data corroborate the findings in this study, because umbelliferone was also found only in the buriti fruit peel. Resveratrol and pterostilbene (doubly methylated form of resveratrol), classified as stilbenes, were identified by Tauchen et al. [24] in the peel and pulp of the buriti fruit. It is noteworthy that, in this study, the compounds were tentatively identified for the first time, in the almond (resveratrol) and the endocarp (pterostilbene). Resveratrol demonstrated a protective effect on oxidative stress biomarkers, preventing lipid peroxidation and stimulating the antioxidant defense system and decreasing brain, heart, liver, and kidney tissue damage in rats [68].

Resveratrol and flavanols (catechins and tannins), which are very common in grape skins, are credited with the beneficial effects of wine consumption, such as cardioprotective, anticancer, antiviral, neuroprotective, antiaging, and anti‐inflammatory effects [69]. Resveratrol and catechin have demonstrated a protective and synergistic effect against *β*‐amyloid toxicity to neurons, strengthening each other's protective effect [70]. Catechin and its derivatives (*m/z* 577, 291, and 307) were found in this study in both ionization modes, mainly in the buriti endocarp.

**FIGURE 3:**
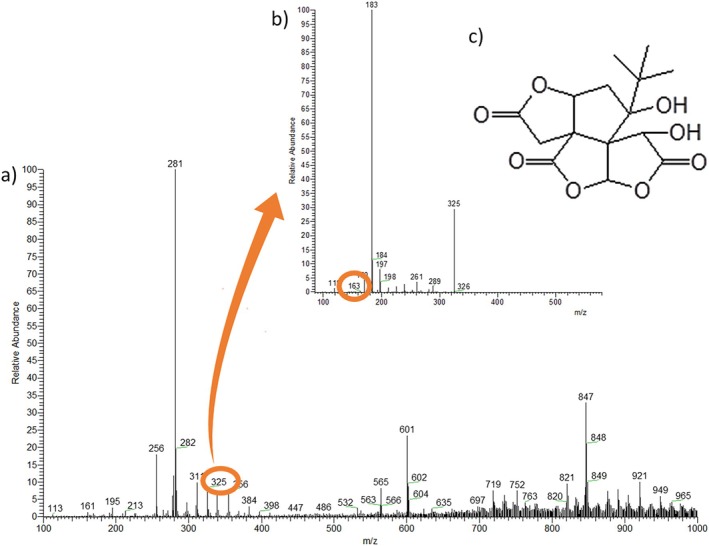
(a) The ion at *m*/*z* 325, when fragmented, produces, among others, the ion at *m*/*z* 163; (b) product ion mass spectrum (MS/MS) of the ion of *m*/*z* 325 [M − H]; and (c) chemical structure of bilobalide.

Among the terpenoids, bilobalide (Figure 3) was detected in several species of the Arecaceae family, according to Lahlou [47]. However, it is the first time that this molecule has been identified in the peel, endocarp, and almond of the buriti fruit. According to these authors, the pulp of *Livistona fulva*, being very rich in phenolics such as bilobalide and kaempferol‐3‐O‐rutinoside, has high antitumor activity due to the synergy between a wide variety of these phenolic compounds. Carnosol has already been reported by Rudke [26] in the almond and the buriti fruit peel; for the endocarp and fruit pulp, it is the first time these compounds are being described.

Bataglion [42] compared GC‐MS and EASI‐MS to obtain a comprehensive lipid characterization of buriti oil and identified that oleic acid (*m/z* 282) was the most abundant compound among the free fatty acids. They also detected, for the first time in buriti pulp oil, stearic acid (*m/z* 284). According to the authors, these findings are valuable for the food, pharmaceutical, and cosmetic industries. Abreu‐Naranjo [1] and de Oliveira [57] observed palmitic acid (*m/z* 255), one of the main components of buriti oil, which was first identified by GC‐MS. Both studies identified the fatty acids in buriti pulp oil, whereas in the positive ionization mode of PS‐MS in this article, it is noteworthy that stearic and oleic acids were detected in the peel and endocarp of buriti for the first time. These authors report that in the positive ion mode, sodium adducts [M + Na] ^+^ were formed during the acquisition of the TAG profile of buriti oil.

It is worth noting that the microdroplets generated in PS (paper spray) contain charged analytes (e.g., protonated, deprotonated, or cation adducts). During the drying process, ionized analytes are transferred to the gas phase, driven by electrostatic repulsion between charges within the droplets [28]. Thus, some observed ions may be in their protonated forms [M + H] ^+^, such as cinnamic acid (*m/z* 149) and apigenin caffeoyl hexoside (*m/z* 595). Adduct formation can also occur, as seen with 5‐deoxyleucopelargonidin (*m/z* 313), which formed a potassium adduct [M + K] ^+^ [28, 48].

## 4 Conclusion

In total, 61 compounds were identified, predominantly in the negative ion mode and of the flavonoid class. Buriti fruit peel stands out in relation to the other fruit parts, with regard to the distribution of fixed constituents, with more than 60% of its compounds being flavonoids. This study presents 26 unpublished compounds, up until this point not reported in studies of buriti fruits. As such, results demonstrate that the fruit has the potential to be fully explored, and further investigations are required regarding the nutritional content of the peel and almond, as well as the biological activity of its bioactive compounds. In this sense, the present study aims to demonstrate that the discarded parts of the buriti fruit have the potential to be introduced to human food. The sensible and integral use of the fruit, in addition to adding value to it and generating income for producers, contributes to the preservation of the Cerrado biome, as well as to reducing waste generation.

## Conflicts of Interest

The authors declare no conflicts of interest.

## Supporting information


**Figure S1** PS (+) MS of methanolic extract of buriti peel
**Figure S2** PS (+) MS of methanolic extract of buriti pulp
**Figure S3** PS (+) MS of methanolic extract of buriti almond
**Figure S4** PS (‐) MS of methanolic extract of buriti peel
**Figure S5** PS (‐) MS of extract methanolic of buriti pulp
**Figure S6** PS (‐) MS of methanolic extract of buriti almond
**Figure S7** Product ion mass spectrum (MS/MS) of the ion of *m/z* 149 [M + H] ^+^ (ascribed as protonated Cinnamic acid).
**Figure S8** Product ion mass spectrum (MS/MS) of the ion of *m/z* 291 [M + H] ^+^ (ascribed as protonated Catechin).
**Figure S9** Product ion mass spectrum (MS/MS) of the ion of *m/z* 307 [M + H] ^+^ (ascribed as protonated Gallocatechin) (‐).
**Figure S10** Product ion mass spectrum (MS/MS) of the ion of *m/z* 313 [M + H] ^+^ (ascribed as protonated 5Deoxyleuco pelargonidin).
**Figure S11** Product ion mass spectrum (MS/MS) of the ion of *m/z* 331 [M + H] ^+^ (ascribed as protonated Malvidine).
**Figure S12** Product ion mass spectrum (MS/MS) of the ion of *m/z* 337 [M + H] ^+^ (ascribed as protonated 3‐O‐Caffeoylshikimic acid (Dactylifric acid)).
**Figure S13** Product ion mass spectrum (MS/MS) of the ion of *m/z* 595 [M + H] ^+^ (ascribed as protonated Rhamnosyl hexosyl luteolin or Apigenin di‐C‐hexoside or Pelargonidine or Apigenin caffeoyl hexoside or Cyanidin‐3‐ rutinoside).
**Figure S14** Product ion mass spectrum (MS/MS) of the ion of *m/z* 609 [M + H] ^+^ (ascribed as protonated Rhamnosyl hexosyl methyl luteolin).
**Figure S15** Product ion mass spectrum (MS/MS) of the ion of *m/z* 611 [M + H] ^+^ (ascribed as protonated Rhamnosyl hexosyl quercetin).
**Figure S16** Product ion mass spectrum (MS/MS) of the ion of *m/z* 625 [M + H] ^+^ (ascribed as protonated Quercetin‐dihexoside).
**Figure S17** Product ion mass spectrum (MS/MS) of the ion of *m/z* 691 [M + H] ^+^ (ascribed as protonated Dihexosyl luteolin sulfate).
**Figure S18** Product ion mass spectrum (MS/MS) of the ion of *m/z* 757 [M + H]^+^ (ascribed as protonated Rhamnosyl dihexosyl luteolin).
**Figure S19** Product ion mass spectrum (MS/MS) of the ion of *m/z* 771 [M + H]^+^ (ascribed as protonated Rhamnosyl dihexosyl methyl luteolin).
**Figure S20** Product ion mass spectrum (MS/MS) of the ion of *m/z* 227 [M ‐ H]^‐^ (ascribed as deprotonated Resveratrol).
**Figure S21** Product ion mass spectrum (MS/MS) of the ion of *m/z* 325 [M ‐ H] ^‐^ (ascribed as deprotonated Bilobalide).
**Figure S22** Product ion mass spectrum (MS/MS) of the ion of *m/z* 335 [M ‐ H] ^‐^ (ascribed as deprotonated 5‐O‐Caffeoyl shikimic acid (neodactylifric acid)).
**Figure S23** Product ion mass spectrum (MS/MS) of the ion of *m/z* 505 [M ‐ H] ^‐^ (ascribed as deprotonated Isoquercetrin acetate).
**Figure S24** Product ion mass spectrum (MS/MS) of the ion of *m/z* 563 [M ‐ H] ^‐^ (ascribed as deprotonated Schaftoside).
**Figure S25** Product ion mass spectrum (MS/MS) of the ion of *m/z* 577 [M ‐ H] ^‐^ (ascribed as deprotonated Catechin/epicatechin dimer).
**Figure S26** Product ion mass spectrum (MS/MS) of the ion of *m/z* 673 [M ‐ H] ^‐^ (ascribed as deprotonated Kaempferol‐3‐O‐sulfate‐4′‐O‐*α*‐rhamnosyl (1 → 6)‐*β*‐d‐glucoside).

## Data Availability

The data that support the findings of this study are available in the Supporting Information.
