# Supplementary material for: Fingerprint Analysis of Buriti ( Mauritia flexuosa ) Using Paper Spray Mass Spectrometry
Source: J Mass Spectrom. 2025 Jul 5;60(8):e5156. doi: 10.1002/jms.5156 (PMC12228054; doi:10.1002/jms.5156)
Supplement: Supplementary file 1 — Figure S1 PS (+) MS of methanolic extract of buriti peel Figure S2 PS (+) MS of methanolic extract of buriti pulp Figure S3 PS (+) MS of methanolic extract of buriti almond Figure S4 PS (‐) MS of methanolic extract of buriti peel Figure S5 PS (‐) MS of extract methanolic of buriti pulp Figure S6 PS (‐) MS of methanolic extract of buriti almond Figure S7 Product ion mass spectrum (MS/MS) of the ion of m/z 149 [M + H] + (ascribed as protonated Cinnamic acid). Figure S8 Product ion mass spectrum (MS/MS) of the ion of m/z 291 [M + H] + (ascribed as protonated Catechin). Figure S9 Product ion mass spectrum (MS/MS) of the ion of m/z 307 [M + H] + (ascribed as protonated Gallocatechin) (‐). Figure S10 Product ion mass spectrum (MS/MS) of the ion of m/z 313 [M + H] + (ascribed as protonated 5Deoxyleuco pelargonidin). Figure S11 Product ion mass spectrum (MS/MS) of the ion of m/z 331 [M + H] + (ascribed as protonated Malvidine). Figure S12 Product ion mass spectrum (MS/MS) of the ion of m/z 337 [M + H] + (ascribed as protonated 3‐O‐Caffeoylshikimic acid (Dactylifric acid)). Figure S13 Product ion mass spectrum (MS/MS) of the ion of m/z 595 [M + H] + (ascribed as protonated Rhamnosyl hexosyl luteolin or Apigenin di‐C‐hexoside or Pelargonidine or Apigenin caffeoyl hexoside or Cyanidin‐3‐ rutinoside). Figure S14 Product ion mass spectrum (MS/MS) of the ion of m/z 609 [M + H] + (ascribed as protonated Rhamnosyl hexosyl methyl luteolin). Figure S15 Product ion mass spectrum (MS/MS) of the ion of m/z 611 [M + H] + (ascribed as protonated Rhamnosyl hexosyl quercetin). Figure S16 Product ion mass spectrum (MS/MS) of the ion of m/z 625 [M + H] + (ascribed as protonated Quercetin‐dihexoside). Figure S17 Product ion mass spectrum (MS/MS) of the ion of m/z 691 [M + H] + (ascribed as protonated Dihexosyl luteolin sulfate). Figure S18 Product ion mass spectrum (MS/MS) of the ion of m/z 757 [M + H]+ (ascribed as protonated Rhamnosyl dihexosyl luteolin). Figure S19 Product ion mass spectrum [file JMS-60-e5156-s001.docx]

**Supplementary Information**

**Figure S1.** PS (+) MS of methanolic extract of buriti peel

**Figure S2.** PS(+)MS of methanolic extract of buriti pulp

**Figure S3.** PS(+)MS of the methanolic extract of buriti almond

**Figure S4.** PS(-)MS of methanolic extract of buriti peel

**Figure S5.** PS(-)MS of the extract methanolic of buriti pulp

**Figure S6.** PS(-)MS of methanolic extract of the buriti almond

**Figure S7.** Product ion mass spectrum (MS/MS) of the ion of *m/z* 149 [M + H]+ (ascribed as protonated Cinnamic acid).


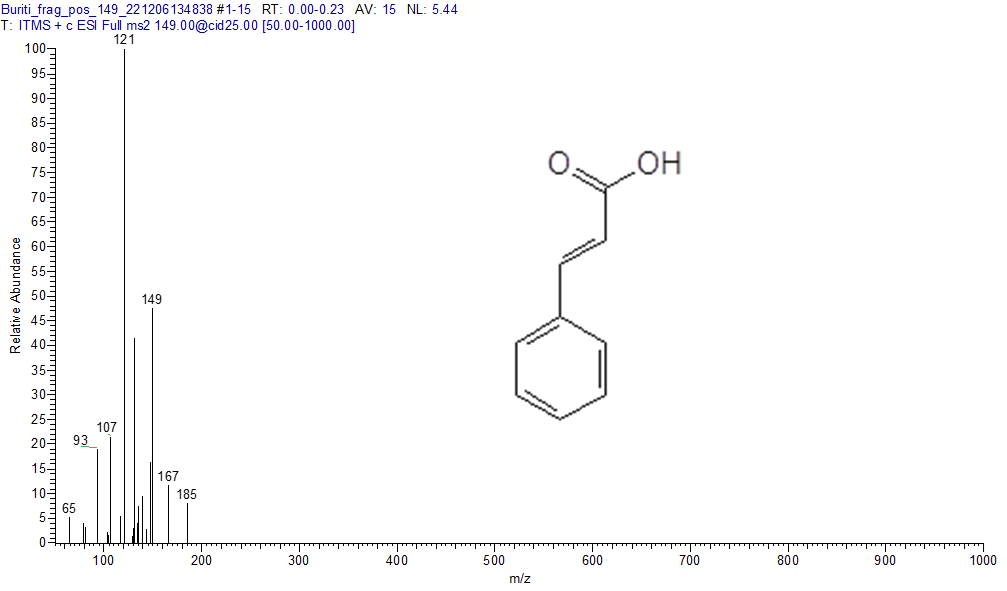


**Figure S8.** Product ion mass spectrum (MS/MS) of the ion of *m/z* 291 [M + H]+ (ascribed as protonated Catechin).


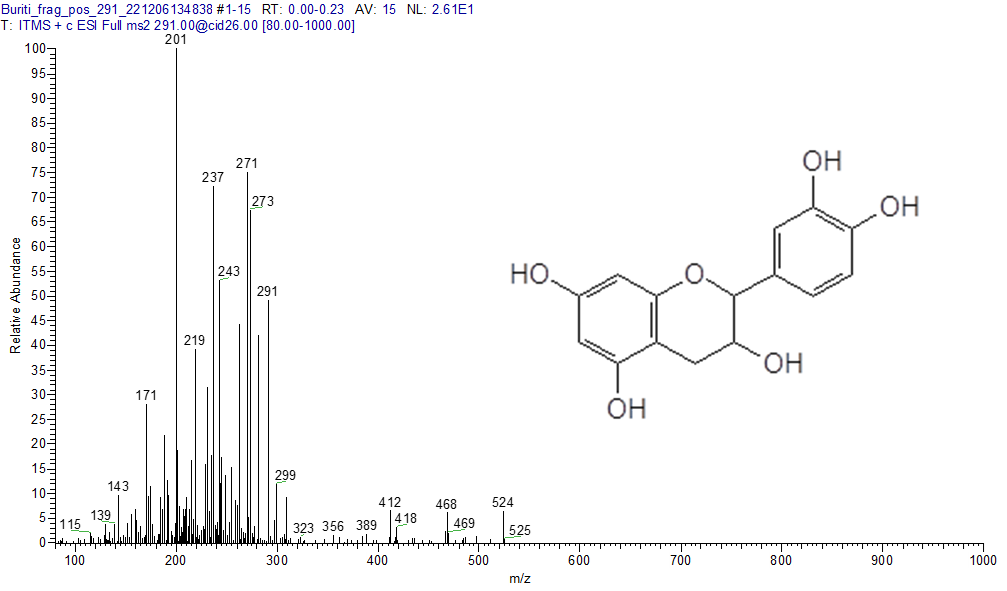


**Figure S9.** Product ion mass spectrum (MS/MS) of the ion of *m/z* 307 [M + H]+ (ascribed as protonated Gallocatechin (-)).


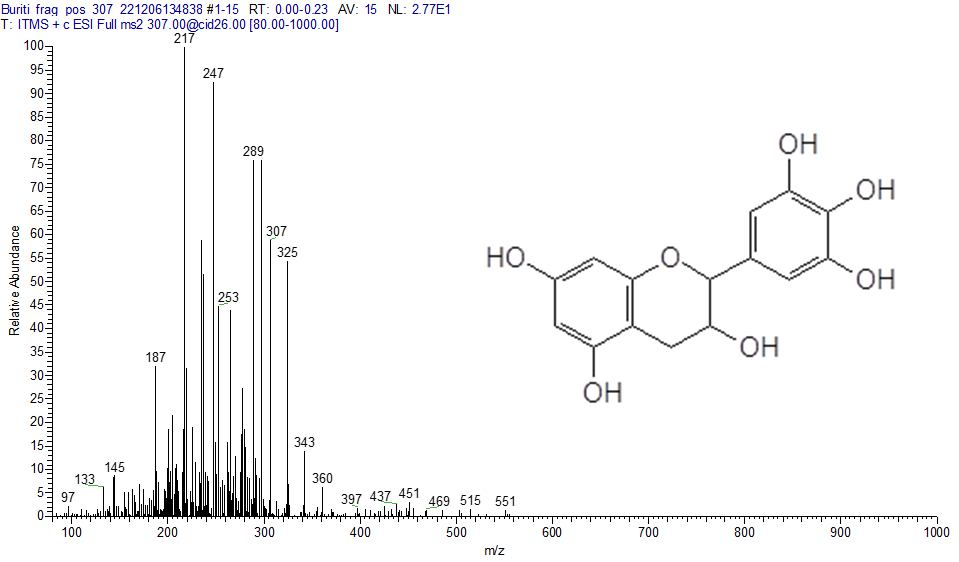


**Figure S10.** Product ion mass spectrum (MS/MS) of the ion of *m/z* 313 [M + H]+ (ascribed as protonated 5Deoxyleuco pelargonidin).


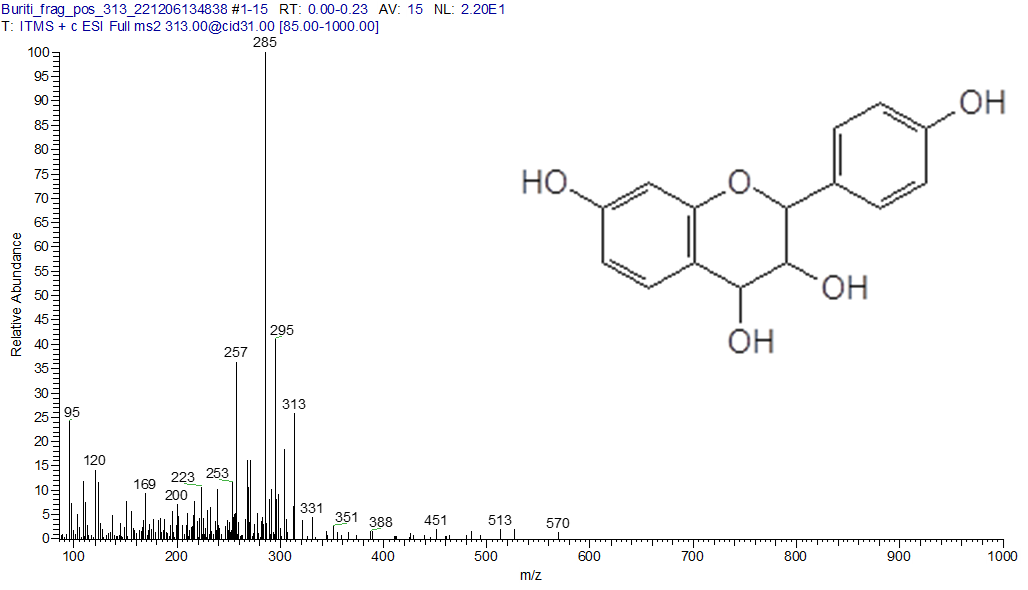


**Figure S11.** Product ion mass spectrum (MS/MS) of the ion of *m/z* 331 [M + H]+ (ascribed as protonated Malvidine).


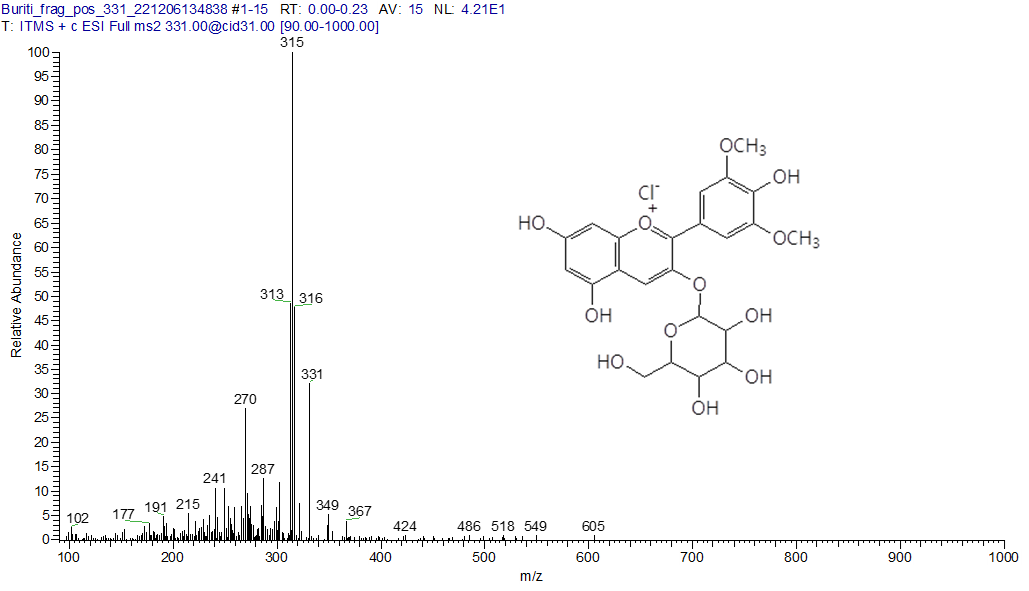


**Figure S12.** Product ion mass spectrum (MS/MS) of the ion of *m/z* 337 [M + H]+ (ascribed as protonated 3-O-Caffeoylshikimic acid (Dactylifric acid)).


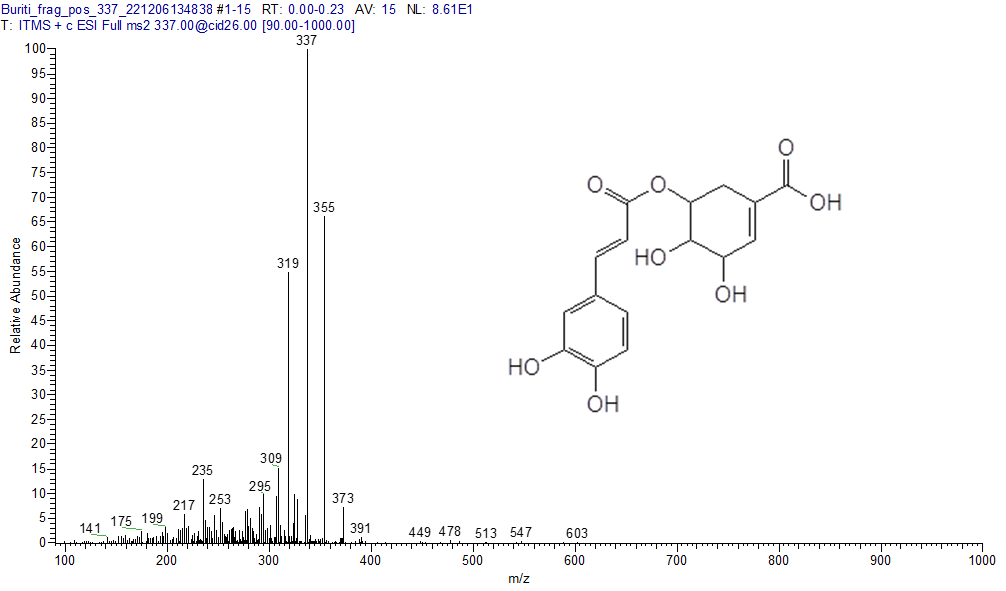


**Figure S13.** Product ion mass spectrum (MS/MS) of the ion of *m/z* 595 [M + H]+ (ascribed as protonated Rhamnosyl hexosyl luteolin or Apigenin di-C-hexoside or Pelargonidine or Apigenin caffeoyl hexoside or Cyanidin-3- rutinoside).


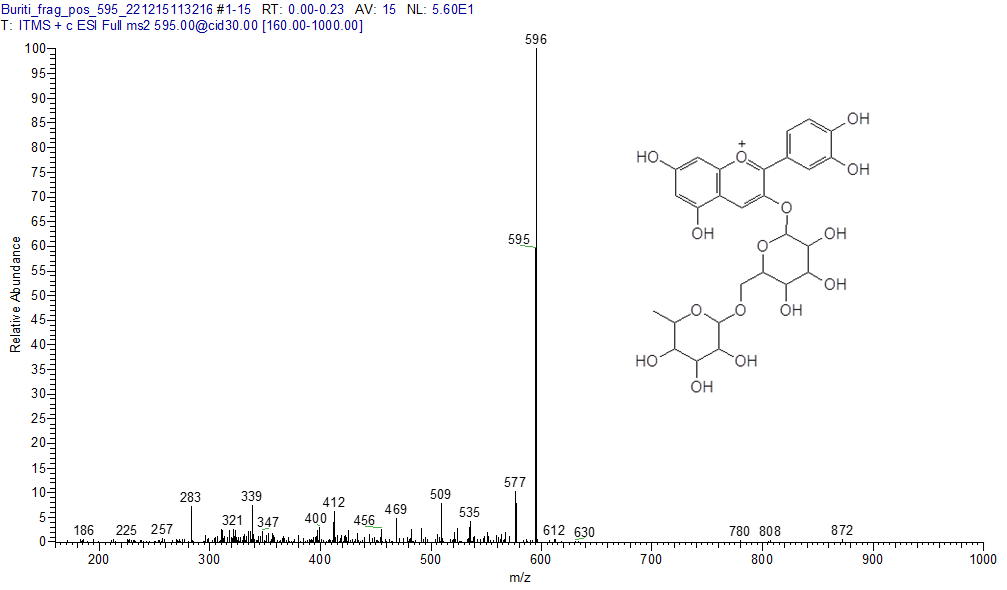


**Figure S14.** Product ion mass spectrum (MS/MS) of the ion of *m/z* 609 [M + H]+ (ascribed as protonated Rhamnosyl hexosyl methyl luteolin).

**Figure S15.** Product ion mass spectrum (MS/MS) of the ion of *m/z* 611 [M + H]+ (ascribed as protonated Rhamnosyl hexosyl quercetin).

**Figure S16.** Product ion mass spectrum (MS/MS) of the ion of *m/z* 625 [M + H]+ (ascribed as protonated Quercetin-dihexoside).


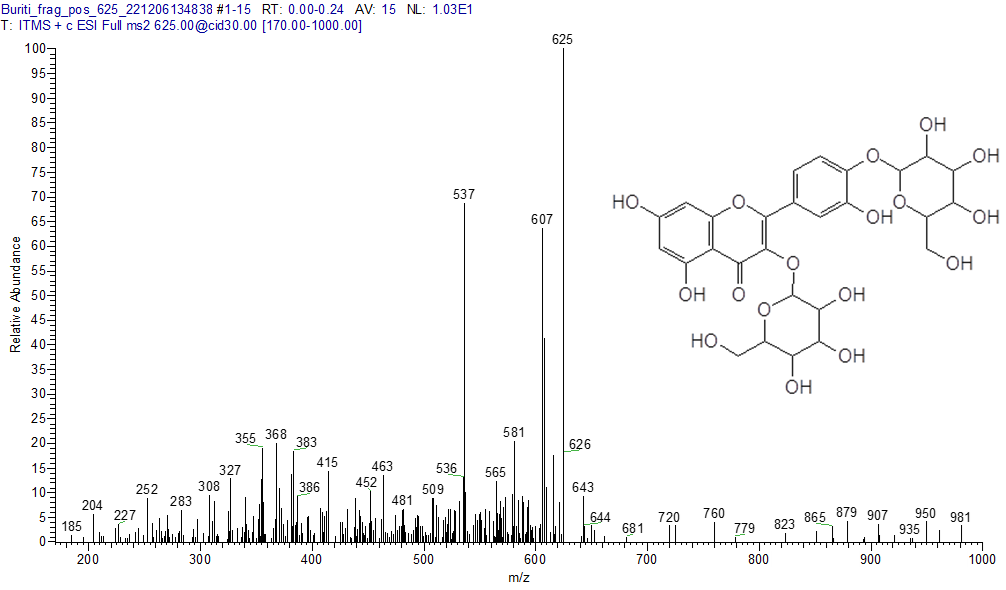


**Figure S17.** Product ion mass spectrum (MS/MS) of the ion of *m/z* 691 [M + H]+ (ascribed as protonated Dihexosyl luteolin sulfate).

**Figure S18.** Product ion mass spectrum (MS/MS) of the ion of *m/z* 757 [M + H]+ (ascribed as protonated Rhamnosyl dihexosyl luteolin).

**Figure S19.** Product ion mass spectrum (MS/MS) of the ion of *m/z* 771 [M + H]+ (ascribed as protonated Rhamnosyl dihexosyl methyl luteolin).

**Figure S20.** Product ion mass spectrum (MS/MS) of the ion of *m/z* 227 [M - H]- (ascribed as deprotonated Resveratrol).


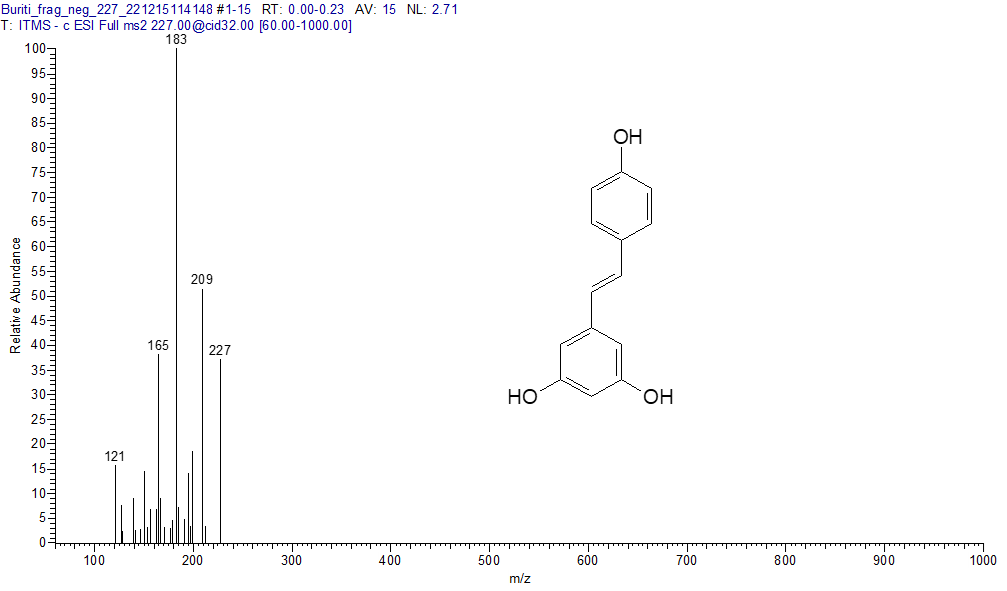


**Figure S21.** Product ion mass spectrum (MS/MS) of the ion of *m/z* 325 [M - H]- (ascribed as deprotonated Bilobalide).


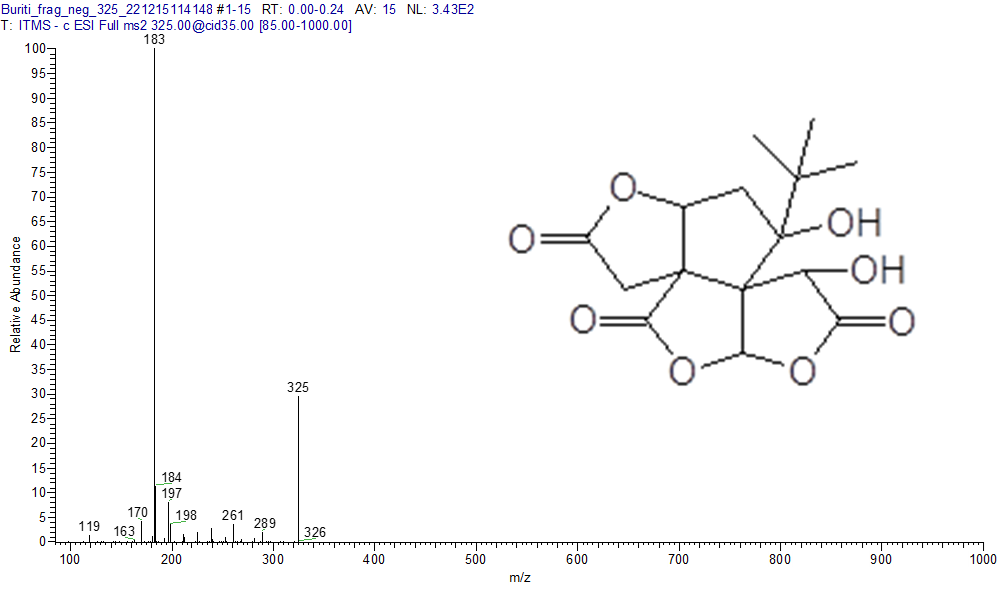


**Figure S22.** Product ion mass spectrum (MS/MS) of the ion of *m/z* 335 [M - H]- (ascribed as deprotonated 5-O-Caffeoyl shikimic acid (neodactylifric acid)).


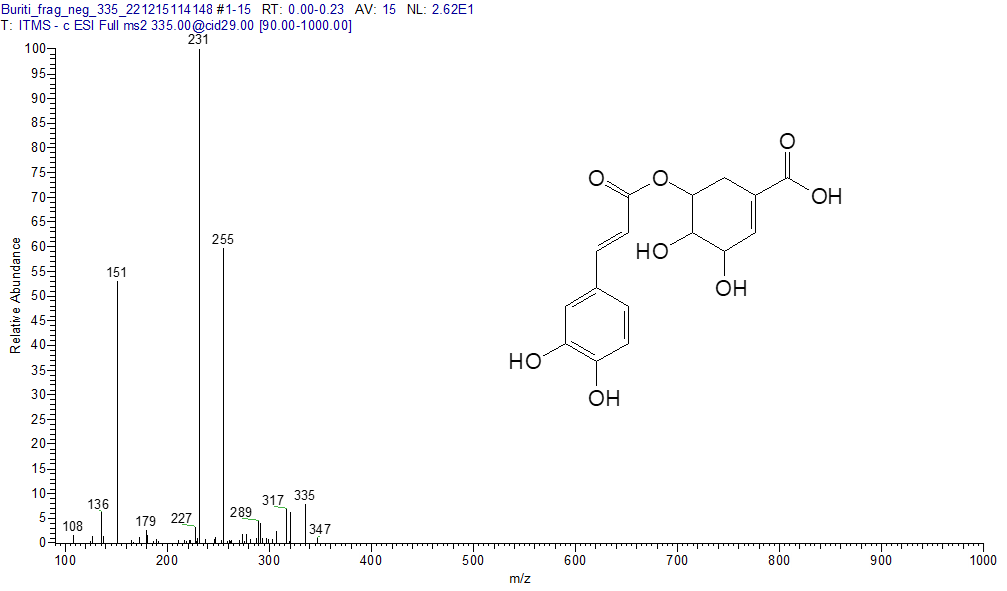


**Figure S23.** Product ion mass spectrum (MS/MS) of the ion of *m/z* 505 [M - H]- (ascribed as deprotonated Isoquercetrin acetate).

**Figure S24.** Product ion mass spectrum (MS/MS) of the ion of *m/z* 563 [M - H]- (ascribed as deprotonated Schaftoside).


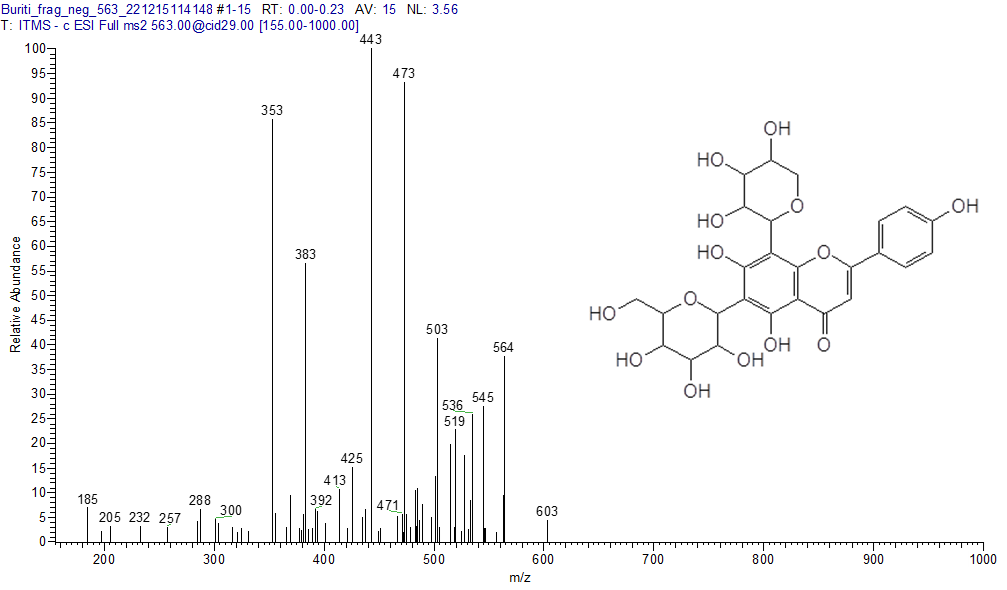


**Figure S25.** Product ion mass spectrum (MS/MS) of the ion of *m/z* 577 [M - H]- (ascribed as deprotonated Catechin/epicatechin dimer).


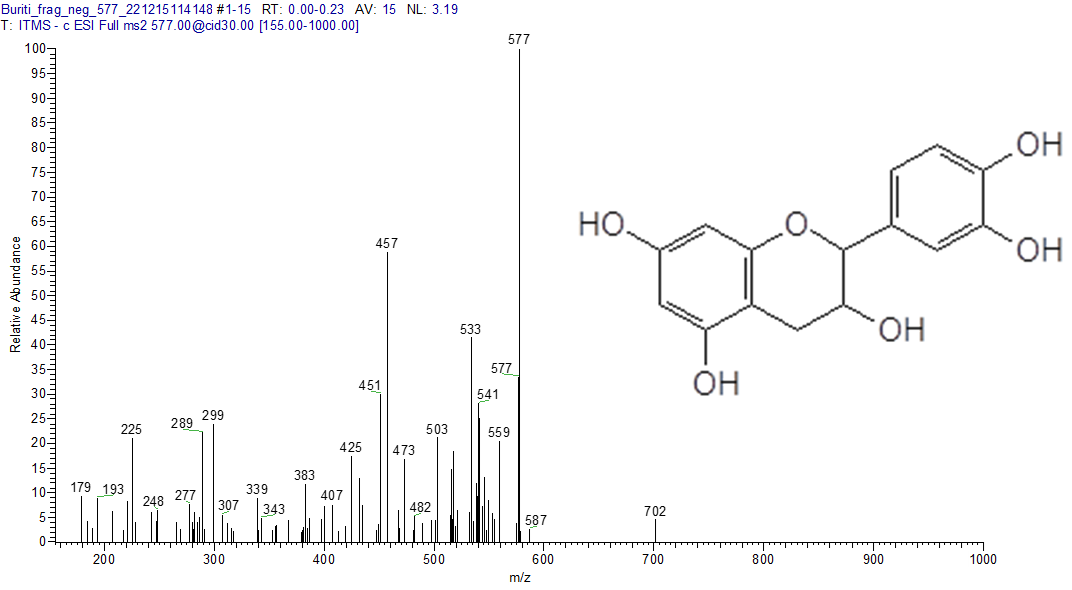


**Figure S26.** Product ion mass spectrum (MS/MS) of the ion of *m/z* 673 [M - H]- (ascribed as deprotonated Kaempferol-3-O-sulfate-4′-O-*α*-rhamnosyl (1→6)-*β*-d-glucoside).
